# Supplementary material for: Leveraging large language models for literature-driven prioritization of protein binding pockets
Source: Bioinformatics. 2025 Aug 7;41(8):btaf449. doi: 10.1093/bioinformatics/btaf449 (PMC12371332; doi:10.1093/bioinformatics/btaf449)
Supplement: btaf449_Supplementary_Data [file btaf449_supplementary_data.docx]

# Leveraging Large Language Models for Literature-Driven Prioritization of Protein Binding Pockets

# Supplementary Information

*Roman Stratiichuk ^1,2^, Mykola Melnychenko ^1^, Ihor Koleiev ^1,3^, Taras Voitsitskyi ^1,3^, Vladyslav Husak ^1,4^, Nazar Shevchuk ^1^, Zakhar Ostrovsky ^1^, Volodymyr Bdzhola ^5^, Semen Yesylevskyy ^1,3,6,7^, Serhii Starosyla ^1^, Alan Nafiiev ^1^.*

^1^ Receptor.AI Inc., 20-22 Wenlock Road, London N1 7GU, United Kingdom.

^2^ Department of Biophysics and Medical Informatics, Educational and Scientific Centre “Іnstitute of Biology and Medicine”, Taras Shevchenko Kyiv National University, 64 Volodymyrska Str., 01601, Kyiv, Ukraine.
^3^ Department of Physics of Biological Systems, Institute of Physics of The National Academy of Sciences of Ukraine, 46 Nauky Ave., 03038, Kyiv, Ukraine.
^4^ Department of Cellular, Computational and Integrative Biology, The University of Trento, Via Sommarive 9, 38123 Povo (Trento), Italy.

^5^ Institute of Molecular Biology and Genetics of The National Academy of Sciences of Ukraine, 150 Zabolotnogo Str., 03143, Kyiv, Ukraine.

^6^ Institute of Organic Chemistry and Biochemistry, Czech Academy of Sciences, CZ-166 10 Prague 6, Czech Republic.

^7^ Department of Physical Chemistry, Faculty of Science, Palacký University Olomouc, 17. listopadu 12, 771 46 Olomouc, Czech Republic.

Table 1. Extraction step prompts before and after optimization.

| Model | Prompts |
| --- | --- |
| Baseline | There is a scientific paper text to analyze indicated by <article> html tags. Do the following:  1. Determine the number of unique binding sites for small molecules in target protein "{target_protein}" that are described in the text.  2. Provide a very laconic, very specific and discriminative characteristic for each binding site that you identified.  3. If the context permits, include any relevant small molecules in the binding site description.  4. Output the list of amino acid residues constituting each of the identified binding sites.  Use the following notation for defining amino acid residues: <chain_id><res_name><res_num>.  4.1 chain_id is an optional chain identifier for each amino acid.  4.2 res_name is the amino acid name in either single-letter or three-letter notation. Use the same notation as in the paper.  4.3 res_num is the integer amino acid number.  4.4 Some residues may be followed by a punctuation mark or a space. Any characters that follow them are not part of the residue. |
| Optimized | There is a scientific paper text to analyze indicated by <article> html tags. Do the following:  1. Determine the number of unique binding sites for small molecules in target protein "{target_protein}" that are described in the text.  2. Provide a very laconic, very specific and discriminative characteristic for each binding site that you identified.  3. If the context permits, include any relevant small molecules in the binding site description, location, and target protein.  4. Output the list of amino acid residues constituting each of identified binding sites. Use the following notation for defining amino acid residues: <chain_id><res_name><res_id>.  4.1 chain_id is an optional chain identifier for each amino acid. Crucially, if the paper specifies a chain ID for a residue (e.g., α, β, γ, A, B, C), you must include that chain ID in the output.  4.2 res_name is the amino acid name in either single-letter (A123) or three letter notation (Ala123). Use the same notation as in the paper.  4.3 res_id is the integer amino acid number.  4.4 Some residues may be followed by a punctuation mark or a space. Any characters that follow them are not part of the residue.  Examples:  Input Text: "Glu89" -- Extracted Residue: {'chain_id: None, res_name: Glu, res_id: 89'}  Input Text: "A134" -- Extracted Residue: {'chain_id: None, res_name: A, res_id: 134'}  Input Text: "GLY1204" -- Extracted Residue: {'chain_id: None, res_name: GLY, res_id: 1204'}  Input Text: "Ala123 ELC" -- Extracted Residue: {'chain_id: None, res_name: Ala, res_id: 123'}  Input Text: "γPhe222" -- Extracted Residue: {'chain_id: γ, res_name: Phe, res_id: 222'}  Input Text: "αY45" -- Extracted Residue: {'chain_id: α, res_name: Y, res_id: 45'}  Input Text: "Tyr101C" -- Extracted Residue: {'chain_id: C, res_name: Tyr, res_id: 101'} |

Table 2. Selected experimental articles in the dataset.

| **#** | **Target protein** | **Paper Name** | **DOI** | **Contains**  **pocket description** |
| --- | --- | --- | --- | --- |
| 1 | Voltage-gated sodium channel from American cockroach | Structure of a eukaryotic voltage-gated sodium channel at near-atomic resolution | 10.1126/science.aal4326 | FALSE |
| 2 | Voltage-gated sodium channel from American cockroach | Structural basis for the modulation of voltage-gated sodium channels by animal toxins | 10.1126/science.aau2596 | TRUE |
| 3 | Sodium channel protein type 7 | Structural basis of Nav1.7 inhibition by an isoform-selective small-molecule antagonist | 10.1126/science.aac5464 | TRUE |
| 4 | Sodium channel protein type 7 | Defining the Functional Role of NaV1.7 in Human Nociception | 10.1016/j.neuron.2019.01.047 | FALSE |
| 5 | GTPase KRas | Identification of MRTX1133, a Noncovalent, Potent, and Selective KRASG12D Inhibitor | 10.1021/acs.jmedchem.1c01688 | TRUE |
| 6 | GTPase KRas | Mega clinical trials which have shaped the RAS intervention clinical practice | 10.1177/1753944716644131 | FALSE |
| 7 | GTPase KRas | Small-molecule ligands bind to a distinct pocket in Ras and inhibit SOS-mediated nucleotide exchange activity | 0.1073/pnas.1116510109 | TRUE |
| 8 | Dihydroorotate dehydrogenase | Regional mapping of the gene encoding dihydroorotate dehydrogenase, an enzyme involved in UMP synthesis, electron transport, and superoxide generation, to human chromosome region 16q22 | 10.1007/BF01232751 | FALSE |
| 9 | Dihydroorotate dehydrogenase | Functional Expression of Human Dihydroorotate Dehydrogenase (DHODH) in pyr4 Mutants of Ustilago maydis Allows Target Validation of DHODH Inhibitors In Vivo | 10.1128/AEM.02569-06 | FALSE |
| 10 | Dihydroorotate dehydrogenase | SAR-Based Optimization of a 4‑Quinoline Carboxylic Acid Analogue with Potent Antiviral Activity | 10.1021/ml300464h | TRUE |
| 11 | Dihydroorotate dehydrogenase | Structures of human dihydroorotate dehydrogenase in complex with antiproliferative agents | 10.1016/s0969-2126(00)00077-0 | TRUE |
| 12 | Dihydroorotate dehydrogenase | Metabolic Modifier Screen Reveals Secondary Targets of Protein Kinase Inhibitors within Nucleotide Metabolism | 10.1016/j.chembiol.2019.10.012 | TRUE |
| 13 | Gamma-aminobutyric acid receptor | Shared structural mechanisms of general anaesthetics and benzodiazepines | 10.1038/s41586-020-2654-5 | TRUE |
| 14 | Gamma-aminobutyric acid receptor | Structural basis of neurosteroid anesthetic action on GABAA receptors | 10.1038/s41467-018-06361-4 | TRUE |
| 15 | 5-hydroxytryptamine receptor 2A | Structure of a Hallucinogen-Activated Gq-Coupled 5- HT2A Serotonin Receptor | 10.1016/j.cell.2020.08.024 | TRUE |
| 16 | 5-hydroxytryptamine receptor 2A | Serotonin 2A (5-HT2A) receptor affects cell-matrix adhesion and the formation and maintenance of stress fibers in HEK293 cells | 10.1038/s41598-020-78595-6 | FALSE |
| 17 | 5-hydroxytryptamine receptor 2A | Structures of the 5-HT2A receptor in complex with the antipsychotics risperidone and zotepine | 10.1038/s41594-018-0180-z | TRUE |
| 18 | Tyrosine-protein kinase ABL1 | Tyrosine Kinase Activity and Transformation Potency of bcr-abl Oncogene Products | 10.1126/science.2408149 | FALSE |
| 19 | Tyrosine-protein kinase ABL1 | Identification and Optimization of Novel Small c-Abl Kinase Activators Using Fragment and HTS Methodologies | 10.1021/acs.jmedchem.8b01872 | TRUE |
| 20 | Tyrosine-protein kinase ABL1 | Discovery and Characterization of a CellPermeable, Small-Molecule c-Abl Kinase Activator that Binds to the Myristoyl Binding Site | 10.1016/j.chembiol.2010.12.013 | TRUE |
| 21 | Tyrosine-protein kinase ABL1 | AMN107 (nilotinib): a novel and selective inhibitor of BCR-ABL | 10.1038/sj.bjc.6603170 | TRUE |
| 22 | DNA polymerase alpha | Mechanism for priming DNA synthesis by yeast DNA Polymerase α | 10.7554/eLife.00482 | FALSE |
| 23 | DNA polymerase alpha | Replicative enzymes, DNA polymerase alpha (pol alpha), and in vitro ageing | 10.1016/j.exger.2003.09.008 | FALSE |
| 24 | DNA polymerase alpha | Structural basis for inhibition of DNA replication by aphidicolin | 10.1093/nar/gku1209 | TRUE |
| 25 | DNA polymerase alpha | Activity and fidelity of human DNA polymerase depend on primer structure | 10.1074/jbc.RA117.001074 | TRUE |
| 26 | Mixed lineage kinase domain-like protein | Insights into the evolution of divergent nucleotide-binding mechanisms among pseudokinases revealed by crystal structures of human and mouse MLKL | 10.1042/BJ20131270 | TRUE |
| 27 | Mixed lineage kinase domain-like protein | ATP-Competitive MLKL Binders Have No Functional Impact on Necroptosis | 10.1371/journal.pone.0165983 | TRUE |
| 28 | Muscarinic acetylcholine receptor M2 | Structure and Dynamics of the M3 Muscarinic Acetylcholine Receptor | 10.1038/nature10867 | TRUE |
| 29 | Muscarinic acetylcholine receptor M2 | Non-Neuronal Functions of the M2 Muscarinic Acetylcholine Receptor | 10.3390/genes4020171 | FALSE |
| 30 | Muscarinic acetylcholine receptor M2 | Muscarinic acetylcholine receptors: novel opportunities for drug development | 10.1038/nrd4295 | TRUE |
| 31 | Muscarinic acetylcholine receptor M2 | Functions of Muscarinic Receptor Subtypes in Gastrointestinal Smooth Muscle: A Review of Studies with Receptor-Knockout Mice | 10.3390/ijms22020926 | FALSE |

Table 3. Selected PDB structures for each target protein.

| **Target protein** | **Uniprot ID** | **PDB** |
| --- | --- | --- |
| Tyrosine-protein kinase ABL1 | P00519 | 2FO0 |
| Voltage-gated sodium channel from American cockroach | D0E0C2 | 6A91 |
| Sodium channel protein type 7 | Q15858 | 5EK0 |
| Dihydroorotate dehydrogenase | Q02127 | 6OC0 |
| Mixed lineage kinase domain-like protein | Q8NB16 | 7MON |
| Gamma-aminobutyric acid receptor | α1 - P14867  α2 - P47869  α3 - P34903  α4 - P48169  α5 - P31644  α6 - Q16445  β1 - P18505  β2 - P47870  β3 - P28472  γ1 - Q8N1C3  γ2 - P18507  γ3 - Q99928  ρ1 - P24046  ρ2 - P28476  ρ3 - A8MPY1  θ - Q9UN88  π - O00591  δ - O14764  ε - P78334 | 6CDU, 6X3T |
| 5-hydroxytryptamine receptor 2A | P28223 | 6A94 |
| DNA polymerase alpha | P09884 | 4QLC |
| GTPase KRas | P01116 | 4DSO |
| Muscarinic acetylcholine receptor M2 | P08172 | 4MQT |

Table 4. Parameter values for the construction of pocket volumetric representation.

| **Parameter name** | **Value** | **Description** |
| --- | --- | --- |
| Threshold chain match | 0.6 | Minimum residue overlap (0.0-1.0) to consider binding site motif matched to the chain (sequence similarity). |
| Threshold cluster match | 0.7 | Minimum residue overlap (0.0-1.0) to consider clusters as a match to the LLM pocket. |
| Threshold merge | 0.7 | Minimum residue overlap (0.0-1.0) required to merge two LLM pockets based on their overlap with Fpocket. |
| Threshold residue match | 0.3 | Minimum percentage (0.0-1.0) of residue atoms in proximity with Fpocket alpha spheres to consider it a contact. |
| Threshold pocket match | 0.1 | Minimum Jaccard index (0.0 - 1.0) for matching LLM-predicted pockets to Fpocket references. |
| Clustering algorithm | MeanShift |  |
| Clustering bandwidth | 10 | Bandwidth parameter for MeanShift clustering. Affects the size/granularity of clusters. |
| Cluster all | False | If True, forces all points into clusters. If False, allows outlier points. |
